# Broad-spectrum resistance against multiple PVY-strains by CRSIPR/Cas13 system in *Solanum tuberosum* crop

**DOI:** 10.1080/21645698.2022.2080481

**Published:** 2022-06-02

**Authors:** Azka Noureen, Muhammad Zuhaib Khan, Imran Amin, Tayyaba Zainab, Nasim Ahmad, Sibtain Haider, Shahid Mansoor

**Affiliations:** a Agricultural Biotechnology Division, National Institute for Biotechnology and Genetic Engineering (NIBGE), A Constituent College of Pakistan Institute of Engineering and Applied Sciences (PIEAS), Faisalabad, Pakistan; b University Institute of Biochemistry and Biotechnology (UIBB), Pir Mehr Ali Shah- Arid Agriculture University, Rawalpindi, Pakistan; c National Centre for Industrial Biotechnology (NCIB), Pir Mehr Ali Shah- Arid Agriculture University, Rawalpindi, Pakistan

**Keywords:** CRISPR/Cas, Indole Acetic acid, PGRs, Potato leaf roll virus, PVY, real-time quantitative PCR, *Solanum tuberosum*, trans-ribo zeatin

## Abstract

*Potato virus Y* (PVY) is a deadly environmental constraint that damages productivity of potato (*Solanum tuberosum*) around the globe. One of the major challenges is to develop resistance against PVY. Emerging clustered regularly short palindromic repeat (CRISPR)/Cas systems have the potential to develop resistance against PVY. In the current research, CRISPR-Cas13 has been exploited to target multiple strains of PVY^N^, PVY^O^, and PVY^NTN^. Multiple genes *PI, HC-Pro*, P3, *Cl1, Cl2*, and *VPg* genes of PVY were targeted by CRISPR/Cas13a. Multiplex gRNA cassettes were developed on the conserved regions of the PVY-genes. Three independent CRISPR/Cas13 transgenic potato lines were developed by applying an optimized concentration of trans-ribo zeatin and indole acetic acid at callus development, rooting, and shooting growth stages. The level of resistance in transgenic plants was confirmed through double-antibody sandwich enzyme-linked immunosorbent assay and real-time quantitative PCR. Our results have shown that efficiency of PVY inhibition was positively correlated with the Cas13a/sgRNA expression. Finding provides the specific functionality of Cas13 with specific gRNA cassette and engineering the potential resistance in potato crop against multiple strains of PVY.

## 1. Background

Potato is the most important consumable non-grain crop. It is the most consumable vegetable crop of 37 million ton production in the year of 2019 around the globe, and production rate of Pakistan was 4.8 million tons (http://www.fao.org/faostat/en/#data/QC). Sustainable potato production is affected by several biotic and abiotic environmental constraints. Among biotic factors, potato viruses like *Potato virus Y* (PVY), *Potato leaf roll virus* (PLRV), *Potato virus A* (PVA), *Potato mop-top virus*, *Potato virus X*, and *Potato virus S* (PVS) are reported. PVY is a significant devastating factor for the potato crop. PVY belongs to the family *Potyviridae* and has multiple strains, and it has affected the tuber quality and yield of crop losses up to 80%.^1^ The PVY genome is single-stranded (ss) positive-sense RNA with approximately 9.7 kb in length. The translated protein 3061 amino acid from the viral genome is processed by viral proteases into a small reading frame (ORF) P1, HC-Pro, P3 (PIPO) 6K1, CI, 6K2, NIa (VPg plus Pro), NIb (viral replicase), and CP (capsid protein).^2^

The mutations in the PVY genomes lead to a higher rate of recombination and diversity that developed an effective mechanism to escape from plant natural immunity. The most significant strains PVY^O^, PVY^N^, PVY^C^, including recombinant strains like PVY^NTN^ and PVY^N:O^ have devastated the potato crop.^3,4^ Previously, several transgenic approaches were applied to develop resistance against PVY, including over-expression of resistance *eIF4E* allele, mutating the susceptible *eIF4E* gene by CRISPR/Cas9, and utilization of RNAi strategy.^5,6^ Furthermore, RNAi approaches have off-target effects due to constitutive siRNA expression.^6,7^ Limitations of several transgenic approaches provided the reasons to develop new strategies for generating broad-spectrum resistance against dominating multiple PVY strains.

The clustered regularly short palindromic repeat (CRISPR)/Cas systems imparted molecular immunity to bacteria, Archaea and invading conjugative plasmid and phages. The immunity attain stepwise 1) acquisition of spacer (gRNA) from invader’s genome, 2) biogenesis and processing of CRISPR RNAs (crRNA), and 3) interference with the invader’s genome.^8,9^ The CRISPR/Cas9, Cas12, and Cas14 have ability to cleave the double stranded^10^ DNA or single-stranded (ss) DNA, while Cas13 has unique ability to cleave the single-stranded (ss) RNA including viral genomes to provide defense.^11,12^

Based on function, the CRISPR/Cas13 belongs to single multi-domain effectors with 900–1300bp range with 4-subtypes (A-D). These subtypes have low sequence homology. Cas13 cleaves the Serna with nonspecific manners with the help of crRNA. Cas13 crRNA possesses a simple structure 20–30 nucleotide guide sequence with a protospacer flanking site of A, U, or C as a preference for the functional activity of Cas13a.^13–15^ The functional activity of Cas13 is greatly affected by the secondary structure of the target sequences. Further, the knockdown of RNA transcript through Cas13a is comparable to the RNA-interference mechanism, but it has reduced off-target effects.^12^ *Lsh*Cas13a has been exploited to gain resistance in monocot grains plant against *Southern rice black-streaked dwarf virus* (SRBSDV) and *Rice Stripe Mosaic Virus* (RSMV).^16^ Potentially, CRISPR/Cas13a system can develop stable resistance against significant RNA viral disease in major crops.^17^ In fields, the plants are exposed to multiple viruses and their strains. More fruitful results would be obtained if conserved regions of multiple viruses or strains would be targeted to attain resistance. The key advantage of using CRISPR/Cas is the simplicity and ease of this system even for targeting multiple sites simultaneously. Multiplexed genome editing (MGE) approach involves more than one gRNA to target multiple regions in the genome. In plants, different strategies have been harnessed to deliver a combination of gRNAs. A construct carrying different gRNAs under the influence of their separate promoters has been used for MGE in various plants, *e.g*. Arabidopsis, maize, wheat, tomato, and rice.^18–21^

In current research, CRISPR/Cas13a has been employed to target the multiple strains of PVY. The response of multiple gRNA cassettes with direct-repeat processing was exploited to develop resistance by targeting conserved coding regions of different PVY strains. We have demonstrated that the CRISPR/Cas13a system can be engineered to confer broad-spectrum resistance in transgenic potato plants against multiple PVY strains. The agrobacterium-mediated transformation protocol was optimized by altering the amount of acetosyringone and plant growth regulators (PGRs) at the time of transformation. The cytokinin (CK) and indole acetic acid (IAA) concentrations were optimized to generate quick stable transgenic lines. The expression of Cas13 has been confirmed by RT-PCR. Functional specificity of CRISPR/Cas13a system was confirmed by treating Cas13 lines with PLRV.

## 2. Results

### 2.1. Construction Preparation

**Figure 1.**
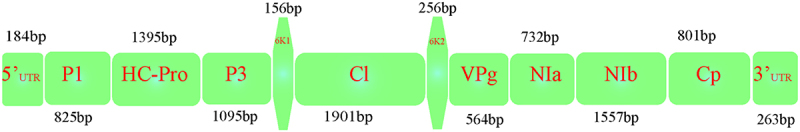
Representation of PVY sequence information. The indication of genes in the PVY genome with respective size.

**Figure 2.**
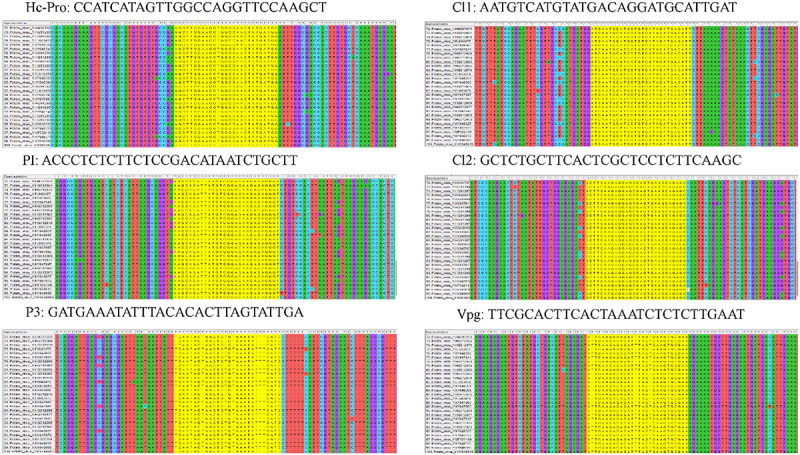
Alignment of 100PVY-sequences to find-out the conserved region through MUSCLES alignment. gRNAs were designed on the conserved regions highlighted as yellow color of the HC-Pro, P1, P3, Cl1, Cl2 and VPggenes of PVY.

**Figure 3.**
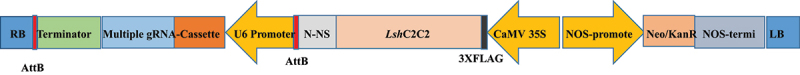
Development of Construct with multiple gRNA cassette and orientation of Cas13 in the map.

The PVY genome has high mutation, recombination rate, and holding genetic diversity among the strains (Fig. 1 and supplementary Table S1).^3^ Alignment of 100 sequences of PVY was conducted using mega muscle alignment tool, based on which gRNAs were designed. A total of six gRNAs were designed based on conserved regions with 28bp sequence and specific PSF-preferences of the following genes as potyviral membrane protein (PI, P3), HC-Pro, cytoplasmic laminate inclusion (Cl, Cl2), and viral genome linked protein (VPg), respectively (Fig. 2, Table 1). Further off-targeting of the gRNA was aligned with the manually against potato whole genome (https://www.plantbreeding.wur.nl/Solyntus/). No off-targets were found with all six targets with 28bp sequence. We constructed the binary vector that harboring the Cas13 (*Lsh*Cas13a, WP_018451595.1) under the CAMV-35S-promoter and sgRNAs and multiplex-sgRNA vector with nuclear export signals expressed under U6-promoter. The construct confirmed through the PCR, restriction analysis, and Sanger sequencing (Fig. 3).

**Table 1.** List of gRNAs.

Gene Name	Sequence
PI	5’ ACCCTCTCTTCTCCGACATAATCTGCTT 3’
HC-Pro	5ʹCCATCATAGTTGGCCAGGTTCCAAGCT 3’
P3	5ʹGATGAAATATTTACACACTTAGTATTGA3’
CI	5’ AATGTCATGTATGACAGGATGCATTGAT3’
CI2	5ʹGCTCTGCTTCACTCGCTCCTCTTCAAGC3’
VPg	5ʹTTCGCACTTCACTAAATCTCTCTTGAAT3’

### 2.2. Confirmation of Multiplex Targeting Construct

**Figure 4.**
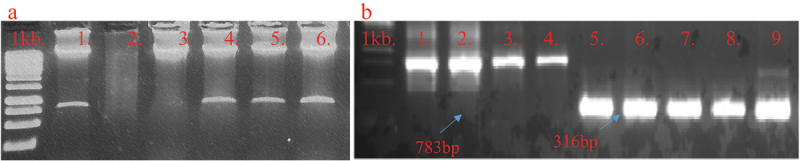
The confirmation of gRNA Cassette in the construct by restricting plasmid through HindIII enzyme and 800 bp elution confirm the gRNA Cassette 4B: Verification of Cas13 and PVY-gRNA Cassette by specific primers.

Multiplex guide RNA cassette was confirmed by restriction analyses. Restriction of plasmid with *Hind III* yielded a product of 792bp (Fig. 4a) indicative of the multiplex gRNA, sequence represented in supplementary data (Supplementary sequence 1, Fig. 1). The restricted product through *HindIII* was ligated into Pk2GW7-LshCas13 vector through restriction-ligation protocols. In the clone, PVY-cassette was confirmed by using specific PCR primers with 783bp product size (Table 2: PVY-cassette confirmation), while Cas13 was confirmed by specific primers that yield a 316bp product size (primer’s sequence in # Table 2 **Cas**13 confirmation Fig. 4b). Final confirmation was carried out by Sanger sequencing.

**Table 2.** Primers for transgene confirmation.

Gene Name	Sequences
Cas13 confirmation	Cas13 F: 5ʹATGGGCAACCTCTTTGGCCATAAGCGTT3’
	Cas13 R: 5ʹCCACTTCTTCAGTCTCGAGGAAATCGTC3’
PVY-Cassette confirmation	PVY-Cassette-F: 5ʹTTTTCTTCTTCTTCGTTCATACAG3’
	PVY-Cassette-R: 5ʹAAAGAAACCAATCGTTGAGAATG3’
Cas13 detection	Cas13-D F:5ʹATGGGCAACCTCTTTGGCCATAAGCGTT3’
	Cas13-D R:5ʹACGAATGTCTATCTCGATCTCCTCC3’
St-Ac	St-AcF1: 5ʹ-GATGGCAGA CGGAGAGGA-3ʹ
	St-AcR1: 5ʹ-GAGGACAGGATG CTCCTC-3ʹ
St-Ac97	St-Ac97F2: 5′-AGTATGACGAATCTGGTCCTTCTATTG-3
	St-Ac97R2: 5′-ACCCAACAATCAACTCTGCCCTCTC-3′
Cas13	Cas13 F: 5’-ATGGGCAACCTCTTTGGCCATAAGCGTT-3’
	Cas13R: 5’- GTACTTGTTTCCGTCGTAGTTGCGCTTC- 3’
HC-Pro	RT-HC1: 5’-ACTCAATGATCCAGTTTTCGAATGCTGA-3’
	RT-HC2: 5’-AACAGATCGCTAACCGGCAAGCTGGCAT-3’
VPg	RT-Vp1: 5’-GAATTCAAGCCTTGAAGTTTCGCCATGC-3’
	RT-Vp2: 5’-TGCGCCCCAGTGAGTGGATCAACGAATT-3’

### 2.3. Transformation of Potato with Optimized PGRs

The internode cuttings from the potato variety Kruda were transformed via *Agrobacterium-*mediated transformation (GV^3101^ strain). Approximately 20 transformed internodes were remained viable on kanamycin selection plate of 25 internodes. The transformed calli were shifted to callus induction medium (CIM) up to 40 days, and healthy surviving calli were transferred to regeneration medium. Regenerated plantlets were moved from shooting medium to rooting medium with 0.2 mg/L and 0.35 mg/L CK and IAA, respectively. Parallel, 4 concentrations of 10 mg/mL, 15 mg/mL, 20 mg/mL, and 30 mg/mL of acetosyringone were added in the simple MS-liquid media at the time of transformation. The 10 mg/mL of acetosyringone has not affected the rate of transformation, while internode treated with 30 mg/mL showed bacterial growth during callus induction stage. The 20 mg/mL of acetosyringone enhanced the transformation efficiency to 10 mg/mL and 30 mg/mL. The optimal AS-treatment duration was 20 min for the internode-transformation (Table 3).

**Table 3.** Concentration of PGRs and media’s composition for optimized tissue culture.

Callus Induction medium (CIM) 1000 mL		3C5ZR medium 1000 mL	
MS salts	4.3 g	MS salts	4.3 g
MSVI Vitamins (stock)	1 mL	3R vitamins (stock)	1 mL
JHMS vitamins (stock)	1 mL	Myo-inositol	100 mg
Myo-inositol	100 mg	Sucrose	30
Sucrose	30 g	IAA (100 mg/mL)	0.5 mL
Trans-zeatin riboside (1 mg/mL)	5 mL	Trans-zeatin riboside (1 mg/mL)	6 mL
IAA (1 mg/mL)		Timentin (100 mg/mL)	3 mL
Agar	4.4	Agar	4.4
pH	5.7	pH	5.7
**Shoot-Propagation/Rooting medium (CM) 1000 ml**		**MS liquid medium 1000 ml**	
MS salts	1 mL	MS salts	4.3 g
Potato vitamins (stocks)	20 g	MSVI vitamins (stock)	1 mL
Sucrose	30 g	JHMS vitamins (stock)	1 mL
Agar	3 g	Sucrose	30 g
IAA (1 mg/mL)	0.35 mg	pH	5.7
Timentin	300 mg		
pH	5.7		
**3R Vitamins (stock) 100 ml**		**JHMS Vitamins (stock) 100 ml**	
Thiamine HCl	100 mg	d-biotin	5 mg
Nicotinic acid	50 mg	Folic acid	25 mg
Pyridoxine HCl	50 mg		
**MSVI vitamins (stock) 100 ml**		**Potato vitamins (stock) 100 ml**	
Glycine	200 mg	Myo-inositol	10 g
Nicotinic acid	50 mg	Thiamanie HCl	40 mg
Pyridoxine HCl	50 mg		
Thiamine HCl	40 mg		

The IAA and Cytokinin (CK trans-ribo zeatin) work antagonistically. We exploited the 100 μL/L, 250 μL/L, 350 μL/L, and 500 μL/L from 100 mg/mL stock of IAA, and 1 mL/L, 2 mL/L, 5 mL/L, and 6 mL/L from 1 mg/mL stock of CK in the plant full MS-media for callus induction (Table 3). We found the best callus development with the application of 350 μL/L and 5 mL/L to IAA and CK, respectively. The application of 0.2 mg/mL CK at shooting-stage provided strong secondary growth.

**Figure 5.**
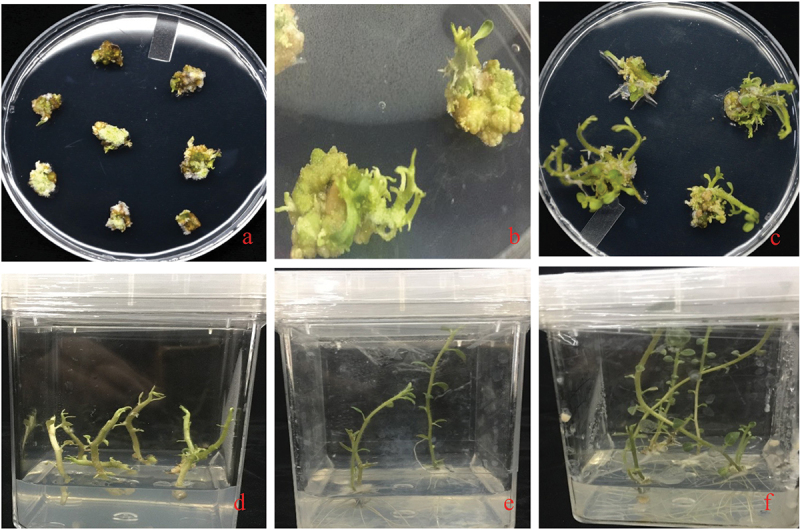
Developmental stages of transformed internodes into plants: The plants internodes were transformed by the Agrobacterium-mediated transformation (GV^3101^). A: the callus development stage, B: Shoot initiation stage C & D: Shoot development stage E&F: Root initiation and development stage.

**Figure 6.**
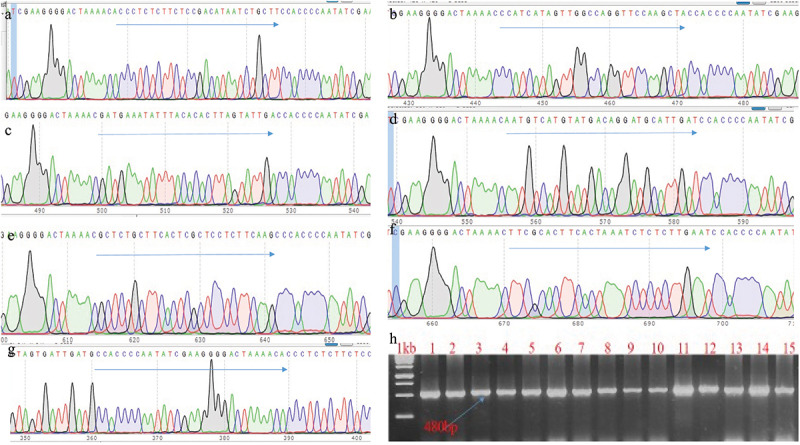
Transgene confirmation and gRNA-component in the plants. Transformed transgenic lines confirmed through PCR and Sanger sequencing to check the presence of gRNA; A is gRNA for the PI, B; HC-Pro, C: P3, D: Cl1, E: Cl2, F: Vpg, G: Direct repeat of Cas13 and H: representing the PCR confirmation of transgenic plants.

The developmental stages from internodes to plants are shown in Fig. 5. The transgenic lines were confirmed by PCR and Sanger sequencing (Fig. 6). The 15 independent transgenic lines were obtained and confirmed by PCR by using Cas13 detection primers (Cas13 F: 5ʹATGGGCAACCTCTTTGGCCATAAGCGTT3’ and Cas13 R: 5ʹCCACTTCTTCAGTCTCGAGGAAATCGTC3’ with 480bp product. To proceed further study, RNA-isolation from these lines and expression of Cas13 were identified. Among them, 3 transgenic lines 13.1, 13.2, and 13.3 were highly expressed Cas13, and these lines were multiplied. Confirmed transgenic lines were shifted to soil and kept in greenhouse under the controlled conditions for establishment of roots growth from media to soil. After 1 month of hardening, the resistance efficiency was verified by phenotypic assay.

### 2.4. PVY-Bioassay and DAS-ELISA

**Figure 7.**
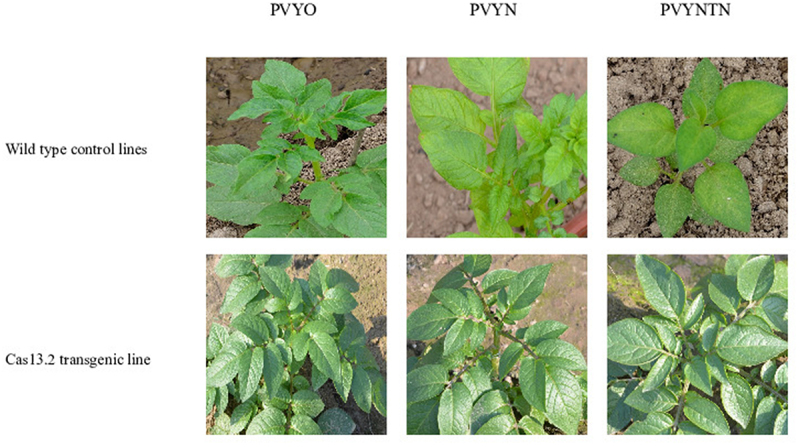
Patho-test against PVY in transgenic and wild control line. Wild-type control plants indicating PVY symptoms in comparison to transgenic lines that representing resistance against PVY strains in the field.

**Figure 8.**
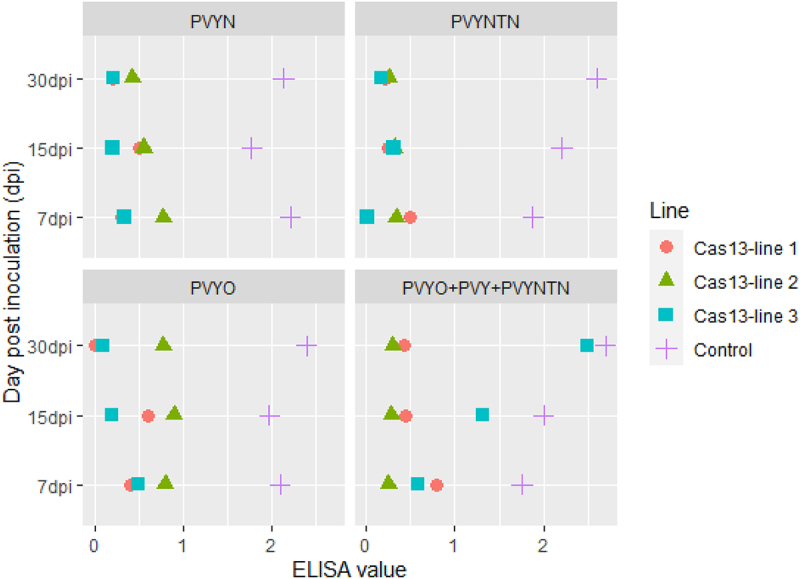
DAS-ELISA values indicate the results of PVY-resistance in three transgenic lines. In the data, line 13.2 is most resistance in response to individual strain and multiple strain infections as compared to other 13.1 and 13.3 lines.

The confirmed strains PVY^O^, PVY^N^, and PVY^NTN^ were applied for the bioassay. To find out the viral load, the bioassay confirmation through DAS-ELISA and real-time qPCR were performed after regular intervals, *i.e*. 7 days post inoculation (dpi), 15 dpi, and 30 dpi. The significant phenotypic symptoms of PVY like mosaic, mottled, and crinkled leaves and vein necrosis were observed in wild-type susceptible control lines (Fig. 7). The three strains of PVY^o^ (HQ912865), PVY^N^ (X97895), and PVY^NTN^ (M95491) were exposed to the transgenic and control lines to check the resistance of the transgenic plants. The resistance assay was checked after regular interval of 7 dpi, 15 dpi, and 30 dpi to monitor the disease symptoms of PVY-strains. Mosaic and vein necrosis were observed in control lines, while no symptoms appeared in transgenic lines Cas13.1, Cas13.2, and Cas13.3, as shown in Fig. 7 and supplementary Figure S2. The data from DAS-ELISA indicated the reduced level of PVY-accumulation in the transgenic lines as compared to the control lines (in graphic chart Fig. 8).

### 2.5. Expression of Cas13 Inversely Proportional to PVY Viral Load

**Figure 9.**
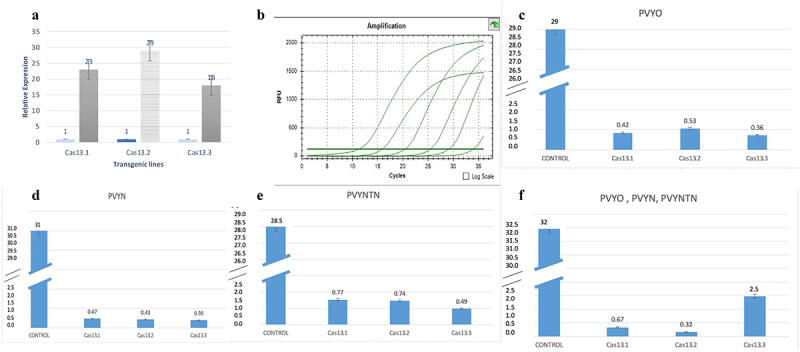
RT-PCR analysis; A; Relative expression of Cas13a in the transgenic lines were represented as compared to control line, error bar is representing the technical repeats. Line Cas13.2 strongly expressing as compared to Cas13.1, Cas13.3. B: Copy number of PVY were determined by qRT-PCR: B; Control dilutions parameters, while C, D, E and F expressing virus titer in the transgenic lines against PVY^O,^ PVY^N^, PVY^NTN^, and co-inoculations of PVY^O,^ PVY^N^, PVY^NTN^ respectively.

To find out the correlation of PVY viral load and Cas13a expression, the RNA-purified from PVY-treated control and transgenic lines were isolated. The cDNA-synthesized real-time qPCR was performed by St-AC and ST-AC97 endogenous primers for *Actin97*. For the Cas13 expression analysis, Cas13 primers were used (Table 2). The expression of *Lsh*Cas13 was observed by the relative RT-qPCR for co-relation between the expression of *Lsh*Cas13 and level of inhibition of PVY-accumulation. The DAS-ELISA and RT-qPCR strongly supported that the higher expression of Cas13 lines holding the higher resistance, as shown in Fig. 9a. The results have shown a positive co-relation of Cas13 expression and inhibition of PVY systemic spreading as crRNA exploited for the multiplexed targeting the conserved regions of the three viral strains. The highest expression of Cas13 was observed in line 13.2. The symptoms observed in this line were near to wild-type untreated control plants. This was also confirmed by the DAS-ELISA where we found strong resistance and no PVY-accumulation was observed. Similar pattern was found in the lines 13.1 and 13.3, where symptoms were moderate that include yellow mosaic pattern. The titer of virus was slightly high as shown by DAS-ELISA and expression of Cas13 was low in Cas13.1 and 13.3. Interestingly, we identified mixed inoculation of PVY^o^, PVY^N^, and PVY^NTN^ on all transgenic lines. Approximately, all lines showed minute to zero viral load, confirmed by RT-PCR by using targeted gene specific primers *HC-Pro* and *VPg* (Table 2). These findings suggest that expression of Cas13 has inverse relation with the symptoms and virus titer. (Fig. 9b–f supplementary Figure S3, Table S2 for additional information).

### 2.6. Identification of Collateral Activity of Cas13

**Figure 10.**
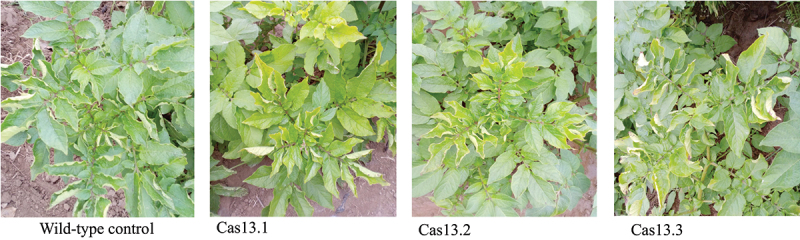
Functional specificity of Cas13 against PLRV. Phenotypic analysis of functional specificity of Cas13 with respect to PVY-targeting gRNA. PVY resistant transgenic lines showed the strong susceptibility against PLRV.

In addition, the CRISPR/Cas13a transgenic lines were treated with PLRV to check the collateral activity of Cas13a. Plants which were inoculated with PLRV showed typical symptoms at 15 dpi that include rolling of the top leaves followed by yellowing (Fig. 10). These symptoms were indistinguishable from that of wild-type control plants. On later stages, plants also showed stunted growth. These plants were subjected to DAS-ELISA for the confirmation, and they showed the presence of PLRV as confirmed through DAS-ELISA. Our results have shown that Cas13a plants failed to provide resistance against heterologous virus, *i.e*. PLRV. Thus, the resistance developed by Cas13a is specific for PVY.

## 3. Discussion

PVY has the potential to devastate the yield of potato crops a large scale. Several conventional approaches have been applied to develop resistance which were met with limited success. Many bacterial and archaeal species protect themselves against invading phages and nucleic acid by activating various forms of Cas proteins to attain immunity. To gain resistance and immunity against DNA and RNA viruses, CRISPR/Cas systems have been widely applied. The enzymes Cas9, Cas12, and Cas14 were applied to target the dsDNA and ssDNA viruses. In case of Cas13a, it cleaves single-stranded (ss) RNA followed by activating and maturation of crRNA and making targeted effector complex.^22^ Inconclusively, Cas13 has the potential ability to develop resistance against ssRNA viruses. In the current study, the CRISPR/Cas13 has been exploited to cleave the ssRNA of multiple strains of PVY as demonstrated in the theoretical model (Supplementary Figure S4).

The Cas13 from *Leptotrichia shahii* (*Lsh*Cas13a) was used to develop resistance against tobacco turnip mosaic RNA virus (TuMV) in model plants *Nicotiana benthamiana* and *Arabidopsis thaliana*. They employed green fluorescent protein fused with TuMV to explore the functional potency of *LshCas13a* against TuMV. Cas13a reduced the expression of HC-Pro more effectively than Coat protein (CP) of TuMV.^23^ Previously, it has been shown that dual and distinct functions of Cas13 for processing the pre-crRNA and degradation of ssRNA moreover, multiple genes can be targeted to develop resistance against pathogens.^24^ In current study, multiple genes, *i.e*. P3, Cl, HC-Pro, and VPg were targeted by Cas13 to develop broad-spectrum resistance. We targeted multiple strains of PVY simultaneously by CRISPR arrays harboring 28-nt targeting the PI, HC-Pro, Cl, and VPg on the conserved regions of the PVY. The gRNAs were assembled with regular interval of 28-nt direct repeats (DRs). These targeted genes are essential for the integrity of viral genome and proper functioning as PI & P3 potyviral membrane protein for the virus replication and systemic spreading and pathogenicity.^1,25^ The CI have been involved in the virus infection and movement.^2,10^ The VPg is essential for interaction with the host-machinery and responsible for the viral genome replication and systemic spreading.^1,26^ In this study, the multiple gRNA-targeting Cas13a harboring vector (14.5kb) has assembled to develop resistance against recombinant strains of PVY.

Recently Desiree and King Edward potato lines were developed against bacterial blight resistance by targeting *DND1* and *DMR6-1* genes at multiple sites.^27^ Transformation efficiencies were improved by optimizing several factors affecting regeneration, including the quality of the starting plant material, acetosyringone, and different concentrations of the PGRs. The plant-derived phenolic compounds work as chemo-attractants for the *Agrobacterium*. The significant phenolics are 3,5 dimethoxyacetophenone (acetosyringone) and hydroxyacetosyringone and act as inducers during Agrobacterium-mediated transformation. The VirA/VirG component of Ti-plasmid senses the acetosyringone as host signals and activates the vir-gene expression.^28^ The transfer and copy number replication of the Ti-plasmid from *Agrobacterium* to plants is regulated by the phenolic signals.^29^ A specific protocol was designed to alleviate and improve the growth vigor and resulted in a 4- to 10-fold increase in transformation efficiency.^30,31^ The phytohormones potentially develop the signaling interaction to regulate several metabolic processes. During the transformation, the IAA and ethylene levels elevated in the potato internodes. The IAA is a substantial requirement for growth and development, while a higher concentration over 50 μM enhanced the suppression of vir-gene that affects the rate of transformation.^32^ The crosstalk between several hormones is a crucial regulatory network in defense reactions against environmental constraints. Previously, it has been shown that a higher level of CK provides immunity and exogenous application of CK increased the level of SA-dependent gene expression during pathogen infection.^33^ CK involve in callus greening and shoot induction in the explant.^34^ Synthetic IAA has been used for root-development, proper leaf growth, and identification of several auxin responsive pathways.^35,36^ IAA and CK play crucial role for regeneration and development.^37^ Basically, the plant’s signals activate the virulence genes of *Agrobacterium* to transfer and integrate the T-DNA to plant nucleus. That expression of foreign T-DNA leads to the expression of IAA and CK. In the current study, we exploited the various amount of PGRs for the optimization, and we noticed the rate of callus development and growth was enhanced by the application of 0.5 mg/L and 0.2 mg/L of trans-ribo zeatin, respectively. We identified that the concentration from 0.1 mg/mL to 0.35 mg/L of IAA promotes the shooting and rooting growth. Upon the induction of acetosyringone, the large size binary vectors significantly improved transformation in potato as previous reported into rice cells.^38^ We observed an improved transformation efficiency with 20 mg/mL of acetosyringone during the transformation of internodes.

The functionality of Cas13a to combat the RNA in the cytoplasm was identified by targeting three different genes in rice by LwaCas13a. Transformed protoplasts exhibited more than 50% knockdown after 48 hours of transformation.^12^ The level of resistance was directly correlated with the expression efficiency of Cas13a. The Lsh-Cas13a was exploited by generating stable transgenic lines to attain resistance against PVY^o^, PVY^NTN^, and PVY^N^. We tested Cas13 lines against three strains of PVY and PLRV to find out the level of resistance, interference, and specificity of Cas13a with guide RNA. Our results are in line with previous studies where CRISPR/Cas13a transgenic *Solanum tuberosum* cv. Desiree was developed having resistance against PVY exhibiting no effects against PVA and PVS.^39^ We stably developed the transgenic plants with multiple gRNAs targeting the conserved regions of the PVY-strains. The multiple gRNA cassette with alternative DRs. These repeats processed as self-cleaving ribozyme,^40^ Csy4 RNase,^27^ or t-RNA processing enzyme,^41^ and Cas13 can process its own pre-crRNA.^22,42^ The latest strategy shows very promising efficiency for MGE in plants and successfully confirmed and provides resistance against multiple recombinant strains of PVY.

In previous studies, the RNA viruses target by Cas9 from *Francisella novicida* (FnCas9) to develop resistance in tobacco and *Arabidopsis*.^43^ Currently, we generated the resistance in tetraploid potato against three strains of PVY. To further test the inheritability of the resistance against PVY^O^, PVY^N,^ and PVY^NTN^ into the successive generations, T2 of the transgenic Cas13 were harvested and would be challenged to various strains of PVY. Overexpressing of crRNA–LshCas13a specifically targeting the viral genome was an effective way to generate stable RNA virus resistance with multiple DRs, which was helpful in over-expression of Cas13a. The qualitative expression of Cas13 was observed in all three resistance lines to check the expression of Cas13 relative to resistance. The RT-PCR indicated the Cas13.3 highly expressed as compared to 13.2 and 13.1 lines. The 13.3 transgenic line exhibited higher resistance against individual PVY^O^, PVY^N^, and PVY^NTN^ assays, while less resistance level was observed when the mixture of strains was applied, which indicates discovering more about the emergence of novel strain or weak expression of gRNA cassette under U6 promoter. The 13.2 line exhibited lowest viral load of PVY in individual treatments and mixed treatment as compared to control, transgenic 13.1, and 13.3 lines. The transgenic line 13.2 possess the higher resistance as compared to transgenic lines 13.1 and 13.3 by qRT-PCR screening assays. Previously reported that the Cas13 have collateral activity after recognition and cleavage of target site, leading to nonspecific degradation of RNA regardless of complementarity to the spacer.^44^ However, these lines failed to show resistance against PLRV. These finding confirm that the resistance produced by Cas13a is highly specific and it does not work against heterologous viruses. PVY-resistant lines have not exhibited collateral activity.

Our finding showed that the RNA-targeting CRISPR/Cas’s system has provided the potential benefits over the DNA genome editing as expanding the functional capacity improving anti-viral immunity and avoiding the pleiotropic effects of genome editing. This research potentially provides new avenues to develop resistance against pathogens especially with RNA genomes.

## 4. Conclusion and Future Prospects

Potato is an important staple food that has a substantial role to feed the burgeoning population of the world. Molecular breeding and genetics have provided tremendous solutions against viral diseases, and the development of CRISPR/Cas systems has speed up the generation and development of resistance. The *Lsh*Cas13 has provided resistance against three strains of PVY simultaneously. This system is widely used for the transcriptome engineering and would be helpful for identification of solutions against ssRNA viruses. Interestingly, the co-evolution of the viruses and microbes propelled the diversification of CRISPR-Cas defense systems to combat novel emerging recombinant strains. The functionality of Cas13 widely exploited in the RNA-base conversions in mammalian cells^45^ that would open the new avenues in the plant editing field. The ability of Cas13 to process pre-crRNA into mature crRNA, which targets the ss RNA of phage genome during viral interference, would be utilized for tracking of editing and signaling pathways in plants.^46^ That eventually helpful in detection of many diseases and exploited in improving crop yield.

## 5. Materials and Methods

### 5.1. Plants, Virus, and Viral Strains

Potato (*S. tuberosum* cultivar Kruda) plants were grown in a growth chamber with conditions mentioned earlier.^47^ The purity of PVY was confirmed by DAS-ELISA. The Local-PVY^o^, PVY^N^, and PVY^NTN^ strains were confirmed by DAS-ELISA (Catalog # V093); most crop damaging strains in Pakistan were used for this study.

### 5.2. Multiplex Designing and Construction of LshCas13a/sgRNA Cassettes

For the development of CRISPR/Cas13a with its multiplex gRNA cassette, specific gRNAs were designed. For this, 100 of PVY (9.7Kb) sequences were aligned by using the Mega-6 muscle alignment software. The multiple 28bp gRNA were chosen on the conserved regions with respect to PFS preferences. The gRNAs targeting the PVY genome were arranged in the Cas13ʹs gRNA Cassette as PI, HC-Pro, P3, Cl1, Cl2, and VPg (Table 1). The gRNAs were manually blast with whole potato genome to identify the off-targets; there were no off-targets found on the potato genome. The multiple gRNAs with alternative 28bp DRs encoded under Arabidopsis U6 promoter; cassette was commercially synthesized. In pK2GW7-pCas13a vector, the Cas13a was expressed under the 35S promoter. pK2GW7-pCas13a vector restricted by *HindIII* and treated with calf intestinal alkaline phosphatase (CIP) to avoid self-ligation and gRNA cassette was restricted from the PTZ57R^kana^ vector by HindIII, specific cassette eluted from gel-purification, and ligated with linear Cas13 vector.

### 5.3. *Transformation of* Solanum Tuberosum *through Agrobacterium*

Constructs harboring the LshCas13a clone with multiple gRNA Cassette were transformed into *Agrobacterium tumefaciens* strain GV^3101^ by electroporation. Briefly, overnight-grown cultures were harvested by centrifugation and suspended in liquid MS-media. The suspension was washed with simple liquid half MS to remove the traces of antibiotics, and final concentrations of O.D_600_, 0.6 was used for potato internodal transformation. Transgenic plants were identified by their resistance to kanamycin.

### 5.4. Optimizing the Transformation Protocols through PGRs at Developmental Stages

The transformation protocol was optimized as 0.6 O. D was measured and culture was pellet down and washed by MS-media to remove the traces of rifampicin and spectinomycin antibiotics. Final O.D for the transformation should be 0.5–0.8 measured and added to the acetosyringone for enhancing the rate of transformation. The concentration of trans-zeatin (naturally CK) and IAA were optimized to get rapid cell division during callus development and early roots and shoot development (Table 3).

### 5.5. Transgene Confirmation

Genomic DNA from transformed *Solanum tuberosum* lines were extracted through CTAB Method.^48^ PCR amplifications were performed to confirm the presence of Cas13 in transgenic control line Cas13.0 without gRNA cassette and transgenic lines with presence of gRNA cassette with Cas13 by specific primers (Table 2). The presence of construct in the transgenic plants was confirmed by Sanger sequencing in three independent transgenic lines 13.1, 13.2, and 13.3.^49^

### 5.6. Mechanical Inoculation of Viruses and DAS-ELISA Assay

Transgenic and wild-type control plants were challenged with the individual and mixed strains of PVY^O^, PVY^N^, and PVY^NTN^. The confirmed transgenic lines were multiplied before shifting to soil. At the 8–10 leaf stages, plants were exposed to viral strain by mechanical rubbing with carborundum powder.^50^ The development of symptoms in transgenic and control lines was analyzed, and samples were collected at the 7-day, 15-day, and 30-day intervals from the inoculated and uninoculated leaves for confirmation of systemic spreading. DAS-ELISA with PVY specific antibodies (Agdia, Elkhart, IN) were performed to analyze the virus accumulation in the inoculated and non-inoculated leaves.^51^

### 5.7. RNA-extraction and qRT-PCR Analyses

The expression of *Lsh*Cas13a and copy number of as determined by of PVY^O^, PVY^N^, and PVY^NTN^ have been analyzed by RT-qPCR assays by using specific primers. Total RNA was isolated by TRIzol reagent^52^ and treated with DNase I (amplification grade, catalog number 18068015). The synthesis of cDNA was carried out by using reverse transcriptase kit (RevertAid First Strand cDNA synthesis Kit: Catalog number: K1622), and oligo (dT) primers. The fold expression of Cas13 was analyzed by qualitative RT-PCR as compared to wild-type control by using Cas13 detection primers in Table 2. PVY titer confirmed by amplification of HC-Pro with 167bp and VPg 176bp and CP 147bp product size with 56°C annealing temperature. Primer sequences for qRT-PCR are listed in Table 2. The technical repeats were performed for each biological replicates. The actin 97 gene was exploited as reference gene.

## Supplementary Material

Supplemental Material

## Figures and Tables

**Table:** 

Cas13a	CRISPR-associated protein
CRISPR	Clustered regularly interspaced short palindromic repeats
CrRNA	CRISPR-RNA
GRNA	Guide RNA
IAA	Indole acetic acid
CK	Cytokinin
ssRNA	Single stranded RNA
PVY	Potato virus Y
PLRV	Potato leaf roll virus
PGRS	Plant growth regulators

## Avalibility of Data and Materials

The materials used in this study would be available from the Dr. Imran Amin (PS); Molecular Virology and Gene Silencing Lab, Agricultural Biotechnology Division (ABD), NIBGE, Faisalabad, Pakistan.
