# Supplementary material for: Broad-spectrum resistance against multiple PVY-strains by CRSIPR/Cas13 system in Solanum tuberosum crop
Source: GM Crops Food. 2022 Jun 2;13(1):97–111. doi: 10.1080/21645698.2022.2080481 (PMC9176253; doi:10.1080/21645698.2022.2080481)
Supplement: Supplemental Material [file KGMC_A_2080481_SM8414.docx]

Supplementary data

Table 1: Sequence and size of PVY whole genome (9.7kb)

| **Gene Name** | **Gene Size** | **Annotation** |
| --- | --- | --- |
| 5′ UTR | 1–184 bp (184 bp) | 5′ untranslated region |
| P1 | 185–1009 (825 bp) | P1 protein |
| HC-Pro | 1010–2404 (1395 bp) | Helper component protease |
| P3^a^ | 2405–3499 (1095 bp) | P3 protein |
| 6K1 | 3500–3655 (156 bp) | 6K1 protein |
| CI^a^ | 3656–5557 (1901 bp) | Cylindrical or cytoplasmic inclusion |
| 6K2 | 5558–5713 (256 bp) | 6K2 protein |
| VPg | 5714–6277 (564 bp) | Viral genome-linked protein |
| NIa | 6278–7009 (732 bp) | First nuclear inclusion protein |
| NIb^a^ | 7010–8566 (1557 bp) | Second nuclear inclusion protein |
| CP^a^ | 8567–9367 (801 bp) | Coat protein |
| 3′ UTR | 9371–9698 (263 bp) | 3′ untranslated region |

Supplementary file 1: gRNA-Cassette sequence

AAGCTTCTTTTTTTCTTCTTCTTCGTTCATACAGTTTTTTTTTGTTTATCAGCTTACATTTTCTTGAACCGTAGCTTTCGTTTTCTTCTTTTTAACTTTCCATTCGGAGTTTTTGTATCTTGTTTCATAGTTTGTCCCAGGATTAGAATGATTAGGCATCGAACCTTCAAGAATTTGATTGAATAAAACATCTTCATTCTTAAGATATGAAGATAATCTTCAAAAGGCCCCTGGGAATCTGAAAGAAGAGAAGCAGGCCCATTTATATGGGAAAGAACAATAGTATTTCTTATATAGGCCCATTTAAGTTGAAAACAATCTTCAAAAGTCCCACATCGCTTAGATAAGAAAACGAAGCTGAGTTTATATACAGCTAGAGTCGAAGTAGTGATTGatgCCACCCCAATATCGAAGGGGACTAAAACACCCTCTCTTCTCCGACATAATCTGCTTCCACCCCAATATCGAAGGGGACTAAAACCCATCATAGTTGGCCAGGTTCCAAGCTACCACCCCAATATCGAAGGGGACTAAAACGATGAAATATTTACACACTTAGTATTGACCACCCCAATATCGAAGGGGACTAAAACAATGTCATGTATGACAGGATGCATTGATCCACCCCAATATCGAAGGGGACTAAAACGCTCTGCTTCACTCGCTCCTCTTCAAGCCCACCCCAATATCGAAGGGGACTAAAACTTCGCACTTCACTAAATCTCTCTTGAATTTGGTTTTGCATTCTCAACGATTGGTTTCAAGCTT

HindIII: AAGCTT

U6-Promoter: Grey colour

gRNA: Highlighted by light orange colour fonts

Direct-Repeats: Black in colour

Terminator: Navy Blue filling


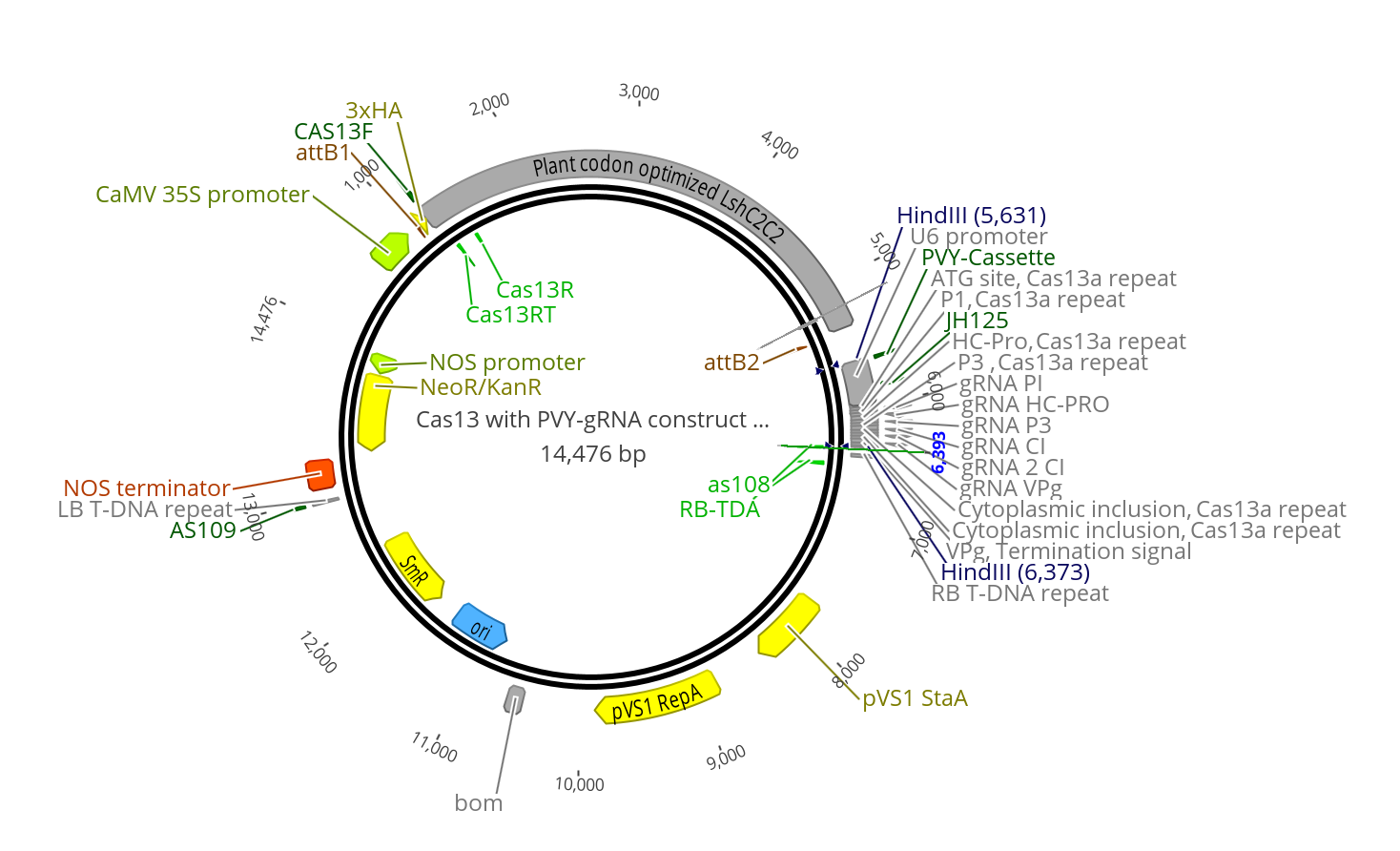


Figure 1: Map of Pk2GW7-Cas13 with gRNA-Cassette

Graphical orientations of Cas13 and gRNA-cassette in the vector.

Figure 2: The PVY’s visual symptoms were appeared in the control lines after 45dpi of infection while all three Cas13-transgenic lines remain resistant against three strains of PVY.

RNA-isolation and quality and RT-PCR

Figure 3: 1: RNA isolation was performed by Trizole reagent 2,3,4: Strandrad for RT-PCR were optimized for measuring the titration of PVY.

Table 2: Serial dilution for RT-PCR

| Sr no | Standard | Concentration (ng/µL) | Concentration in scientific notation | Copy number |
| --- | --- | --- | --- | --- |
| 1 | S2 | 0.05 | 5.000E-02 | 2.3.E+08 |
| 2 | S3 | 0.005 | 5.000E-03 | 2.3.E+07 |
| 3 | S4 | 0.0005 | 5.000E-04 | 2.3.E+06 |
| 4 | S5 | 0.00005 | 5.000E-05 | 2.3.E+05 |
| 5 | S6 | 0.000005 | 5.000E-06 | 2.3.E+04 |
| 6 | S7 | 0.0000005 | 5.000E-07 | 2.3.E+03 |


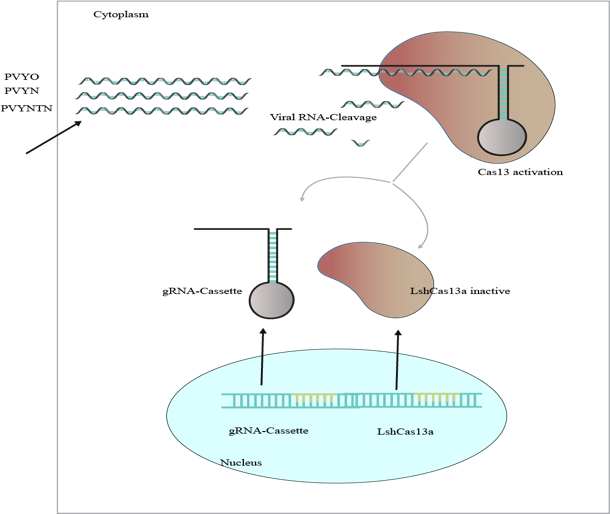


Figure 4: It is model for the representation of PVY replication in cytoplasm. Cas13 and gRNA-Cassette transcribed from nucleus to cytolplasm and Cas13 activated upon interaction with gRNA and DRs. The funcational active unit CRISPR/Cas13a cleave the multiple strains of PVY at specific site directed by gRNA.
